# Iron Homeostasis in Bacillus subtilis Requires Siderophore Production and Biofilm Formation

**DOI:** 10.1128/AEM.02439-18

**Published:** 2019-01-23

**Authors:** Adrien Rizzi, Sébastien Roy, Jean-Philippe Bellenger, Pascale B. Beauregard

**Affiliations:** a Centre SÈVE, Département de chimie, Faculté des Sciences, Université de Sherbrooke, Sherbrooke, Canada; b Centre SÈVE, Département de biologie, Faculté des Sciences, Université de Sherbrooke, Sherbrooke, Canada; Chinese Academy of Sciences

**Keywords:** *Bacillus subtilis*, biofilm, iron homeostasis

## Abstract

Iron (Fe) is the most important metal in biology. Despite its abundance, Fe is mostly present as a ferric form in soils, strongly limiting its bioavailability. To overcome the challenge of Fe acquisition, many microorganisms produce siderophores to retrieve Fe from natural sources. Another ubiquitous feature of bacteria in natural environments is biofilm formation. Previous studies showed that external Fe strongly influenced biofilm formation in several bacteria, suggesting that this microenvironment plays a mechanistic role in micronutrient acquisition for bacteria. Here, we applied a complementary set of analytical methods and deletion mutants to evaluate the role of biofilm formation, siderophore production, and their interaction in Fe homeostasis in Bacillus subtilis. We observed that Fe homeostasis, i.e., active growth at a constant intracellular Fe concentration, requires both siderophore production and biofilm formation. Also, we report that in B. subtilis, both biofilm formation and siderophore production are required to achieve active Fe acquisition from the medium and to sustain normal growth. Furthermore, we provide evidence that the formation of biofilm slightly enhances the kinetics of Fe complexation by catechol siderophores and markedly improves siderophore use efficiency. These results provide new perspectives on the mechanism underlying siderophore-based acquisition of Fe in biofilm-forming bacteria.

**IMPORTANCE** Iron acquisition is of fundamental importance for microorganisms, since this metal is generally poorly bioavailable under natural conditions. In the environment, most bacteria are found tightly packed within multicellular communities named biofilms. Here, using the soil Gram-positive bacterium Bacillus subtilis, we show that biofilm formation and the production of siderophores, i.e., small molecules specifically binding metals, are both essential to ensure Fe uptake from the medium and maintain cellular Fe homeostasis. The biofilm matrix appears to play an important role favoring the efficient usage of siderophores. Taken together, our results demonstrate a close link between biofilm formation and iron acquisition in B. subtilis, allowing a better comprehension of how bacteria can cope with metal limitation under environmental conditions.

## Abstract (precis)

Iron acquisition is of fundamental importance for microorganisms, since this metal is generally poorly bioavailable under natural conditions. In the environment, most bacteria are found tightly packed within multicellular communities named biofilms. Here, using the soil Gram-positive bacterium Bacillus subtilis, we show that biofilm formation and the production of siderophores, i.e., small molecules specifically binding metals, are both essential to ensure Fe uptake from the medium and maintain cellular Fe homeostasis. The biofilm matrix appears to play an important role favoring the efficient usage of siderophores. Taken together, our results demonstrate a close link between biofilm formation and iron acquisition in B. subtilis, allowing a better comprehension of how bacteria can cope with metal limitation under environmental conditions.

## INTRODUCTION

Iron (Fe) is one of the most important metals for all living organisms (1) and one of the most abundant metals in Earth's crust and soils (2, 3). However, under oxic conditions, Fe is predominantly present as hydroxide and oxide forms (4). Common Fe oxides and hydroxides are characterized by very low solubility, not greater than 10^−18^ M at pH 7 (5), and slow dissolution kinetics (6). Most terrestrial organisms thus live in an Fe-rich environment with low Fe bioavailability. To solve this conundrum, organisms have developed different strategies to promote Fe acquisition from their surroundings (7). One of these strategies is the production of low-molecular-weight chelating agents with a high affinity for Fe, named siderophores (8). Siderophores, whose production is controlled by intracellular Fe deficiency, can enhance Fe availability by facilitating the dissolution of Fe oxides and competing with natural Fe complexes (9, 10).

Another important feature of most soil microorganisms is their capacity to form multicellular communities embedded in a self-secreted extracellular matrix, known as biofilms (11). Biofilms are ubiquitous in the environment and provide many advantages for the microbial communities, such as protection against environmental stresses and increased sharing of resources. Additionally, the extracellular biofilm matrix exhibits several chemical properties, which vary as a function of its structure and chemical composition. For example, biofilm matrix can sorb organic and inorganic compounds (12–14). Electron donor or acceptor activities have also been reported in certain biofilm matrices (12).

Biofilms from a large variety of bacteria, such as Bacillus subtilis (15), Pseudomonas aeruginosa (16–18), and Staphylococcus aureus (19), were shown to be regulated by the environmental concentration and chemical form of Fe. In P. aeruginosa, intracellular Fe status was suggested to play a significant role in biofilm development through Fur, a conserved iron-binding transcription regulator (16). The impairment of siderophore production via mutagenesis, or the addition of exogenous siderophores and other Fe-chelating agents in the culture medium, can decrease biofilm formation by P. aeruginosa (20). In B. subtilis, an increased concentration of extracellular FeCl_3_ strongly promotes biofilm formation (15), and deletion of the genes encoding the biosynthesis of the siderophore bacillibactin affects complex colony development (21). Recently, the presence of exogenous siderophores was reported to promote sporulation in B. subtilis, a cellular pathway controlled by the same transcriptional regulator as biofilm formation (22). The sum of these observations not only clearly shows that biofilm formation and Fe acquisition are closely related but also suggests that biofilm formation might play an important mechanistic role in the siderophore-assisted acquisition of Fe in biofilm-forming bacteria.

Here, we applied a complementary set of analytical methods and deletion mutants to study how biofilm formation and siderophore production interact to support Fe homeostasis and growth in the model bacterium Bacillus subtilis. Our data indicate that in B. subtilis, both biofilm formation and siderophore production are essential to sustain Fe acquisition and Fe homeostasis. Our data also show that the kinetics of Fe complexation by catechol siderophores is slightly improved in the presence of biofilm. More importantly, we report that the presence of a biofilm significantly increases the acquisition of Fe-siderophore complexes. These results provide new perspectives on the mechanism underlying Fe acquisition by biofilm-forming bacteria.

## RESULTS

### Biofilm formation, siderophore production, and Fe homeostasis.

To examine how biofilm formation and siderophore production influence Fe homeostasis, we first characterized intracellular Fe concentrations, siderophore (bacillibactin and DHBA [2,3-dihydroxybenzoic acid]) production, and biofilm induction during the growth of B. subtilis in the presence of 10^−4^ M FeCl_3_. We used a B. subtilis strain bearing the biofilm reporter P*_tapA_-yfp*, a transcriptional fusion between the promoter of the matrix gene *tapA* and the gene encoding yellow fluorescent protein (YFP). This construction was introduced at the *amyE* locus, and consequently, the expression of YFP is concomitant with matrix gene expression but does not interfere with it. The expression of the matrix gene can be followed by evaluating the percentage of YFP-positive cells in a population by using flow cytometry (see Materials and Methods for details). Because of cell clumping and matrix production, cell growth in a biofilm cannot be efficiently monitored using optic density or cell enumeration techniques. Thus, we used cellular phosphorus quantitation as a proxy for cell quantification (see Fig. S1 and methods in the supplemental material).

**FIG 1:**
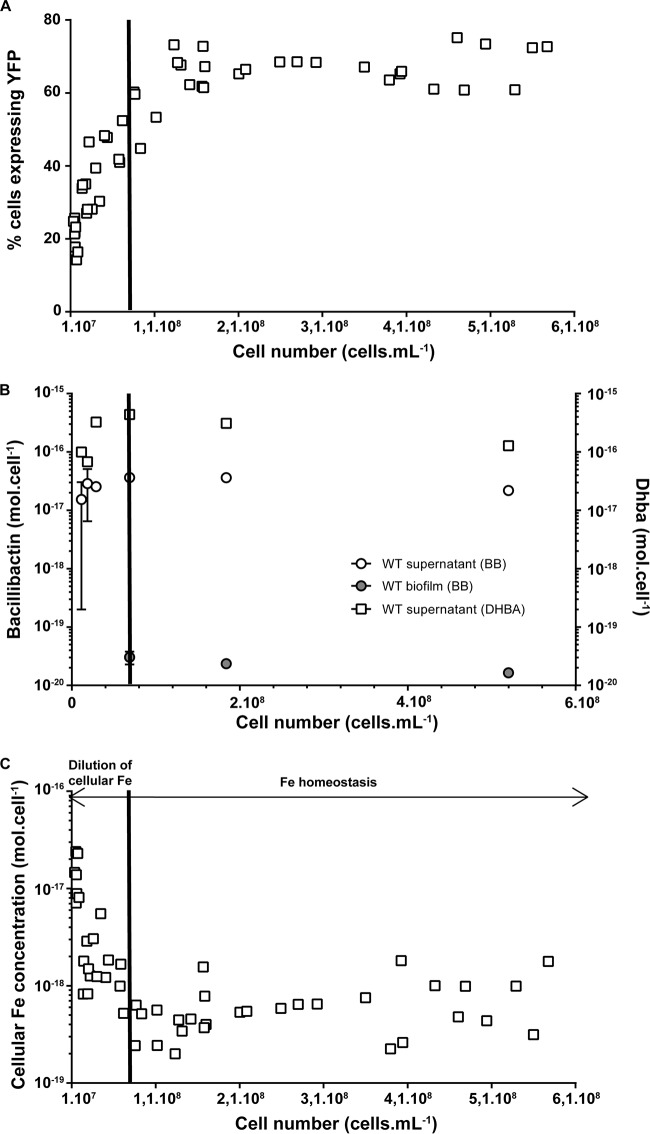
Biofilm induction, siderophore production, and cellular Fe quotas during the growth of B. subtilis 3610 P*_tapA_-yfp* grown at 30°C in a static MSgg medium supplemented with 10^−4^ M FeCl_3_. (A) P*_tapA_-yfp* induction; phosphorus quantification was used as a proxy for cell quantification (see Fig. S1 in the supplemental material). (B) Siderophore production (circles represent bacillibactin production and squares are for the production of DHBA). (C) Intracellular Fe concentration. Results are the means from the three biological replicates, and error bars represent standard deviations.

As shown in Fig. 1A, the YFP reporter—and thus, biofilm formation—was expressed when cells reached a density of approximately 3 × 10^7^ to 5 × 10^7^ cells · ml^−1^, corresponding to 19 to 20 h after inoculation. Our measurements of bacillibactin and DHBA concentrations showed that their amounts were relatively constant (2.9 × 10^−17 ^ mol · cell^−1^ ± 4.3 × 10^−18 ^mol · cell^−1^ and 5.6 × 10^−16^ mol · cell^−1^ ± 1.4 × 10^−17^ mol · cell^−1^, respectively) throughout growth (Fig. 1B). Since preculture was performed on a complex medium (Lennox broth [LB]), our analyses of intracellular Fe levels demonstrate that cells contained large amounts of intracellular Fe, likely exceeding minimum physiological needs, in the hours following inoculation (Fig. 1C). In the early stage of growth (from 13 to 15 h, up to a density of 2 × 10^7^ cells · ml^−1^) (see Fig. S2), bacterial cells were actively producing siderophores but not biofilm (Fig. 1A and B). Despite this active production of siderophores, we observed a steady decrease in intracellular Fe concentrations with increasing cell numbers. However, this sharp decrease in intracellular Fe levels stopped at the mid stage of growth (∼19 to 22 h; 5 × 10^7^ to 1 × 10^8^ cell density · ml^−1^) despite active growth, showing that cells had reached a homeostasis equilibrium for Fe (i.e., active bacterial growth at constant intracellular Fe concentration of ∼4 × 10^−18^ mol_Fe_ · cell^−1^). This stabilization in intracellular Fe concentrations corresponded to biofilm formation, suggesting that the presence of both siderophores and biofilm is required to support Fe homeostasis.

### Siderophores and biofilm are both required to sustain bacterial growth and Fe acquisition.

**FIG 2:**
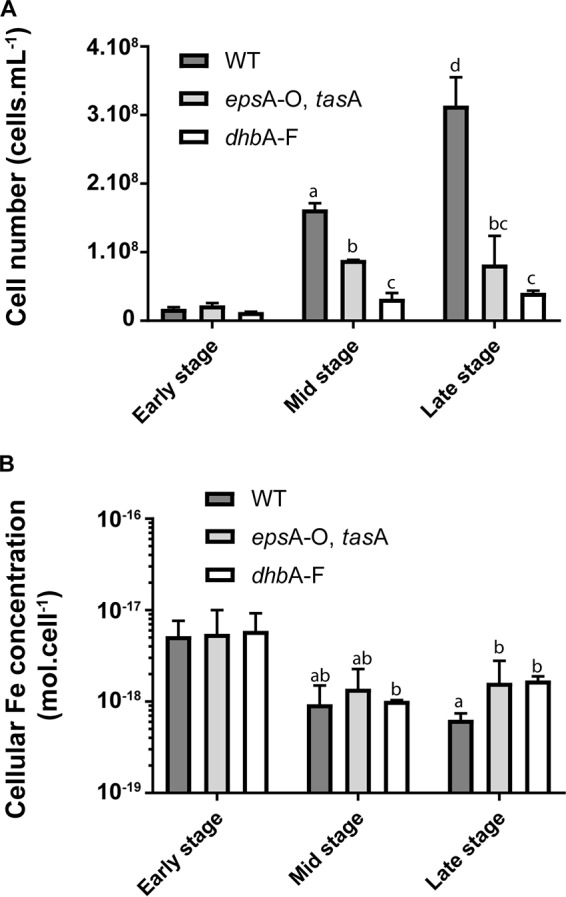
Effects of siderophore production and biofilm formation on bacterial growth and intracellular Fe concentration. Bacterial density (A) and intracellular Fe concentrations (B) of B. subtilis WT, *epsA-O tasA*, and *dhbA-F* strains. Phosphorus quantification was used as a proxy for cell quantification (Fig. S1). Results are the means from the three biological replicates, and error bars represent standard deviations. Strains were grown at 30°C in MSgg medium supplemented with 10^−4^ M FeCl_3_. Lowercase letters indicate significant differences (*P* < 0.05).

To further examine the importance of siderophore production and biofilm formation for B. subtilis Fe homeostasis under our conditions, we compared the growth of B. subtilis wild type with the growth of two mutants, one deficient for biofilm formation (with deletions of the *epsA-epsO* operon and the *tasA* gene [*epsA-O tasA*]) and the other deficient for siderophore production (with a deletion of the *dhbA-dhbF* operon [*dhbA-F*]), in MSgg medium supplemented with 10^−4^ M FeCl_3_. As shown in Fig. 2A, the growth of both mutants was significantly decreased compared to that of the wild type. Indeed, wild-type cells achieved significantly higher cellular densities at mid and late stages of growth in static liquid MSgg than both the *epsA-O tasA* and *dhbA-F* mutants. These low cellular densities for the mutants reflect low growth rates, since wild-type (WT) cells achieved a higher exponential growth rate (μ = 0.317 ± 0.017 cell · h^−1^) for a longer period of time (up to 27 h) than both the *epsA-O tasA* and *dhbA-F* mutants (μ = 0.211 ± 0.015 cell · h^−1^ and 0.197 ± 0.007 cell · h^−1^ up to ∼23 h for the *epsA-O tasA* and *dhbA-F* mutants, respectively) (see Fig. S3). The growth rates of the WT were not significantly different before and after biofilm induction (∼19 h), while for both mutants, bacterial growth was drastically reduced after ∼23 h (μ < 0.03 and 0.08 cell · h^−1^ for the *epsA-O tasA* and *dhbA-F* mutants, respectively). Importantly, the *epsA-O tasA* mutant exhibited growth identical to that of the wild type under nonbiofilm conditions (in MSgg with shaking at 150 rpm) (see Fig. S4), showing that this growth defect is solely associated with biofilm formation and not another feedback pathway. Taken together, these results strongly suggest that both siderophore production and biofilm formation are required to support normal bacterial growth in static liquid MSgg.

Since the siderophore-deficient and the biofilm-deficient mutants displayed impeded growth in an iron-rich medium (10^−4^ M FeCl_3_), we examined if this phenotype was related to Fe homeostasis. In wild-type cells, the rate of decrease in intracellular Fe was consistent with the rate of cell division, suggesting that cellular Fe stocks significantly contribute to growth during the early stage of growth (Fig. 1 and 2). Such consumption of intracellular Fe stocks might explain the active growth of the mutant strains in the early stage of growth (compare Fe cellular concentrations between early and mid stages for the three strains) (Fig. 2B). At mid stage, all strains reached similar intracellular Fe concentrations (∼4 × 10^−18^ mol_Fe_ · cell^−1^). This value is close to the Fe requirement for heterotrophic bacteria reported by Tortell et al. (1.55 × 10^−18^ to 2.16 × 10^−18^ mol_Fe_ · cell^−1^ [23]). However, wild-type cells producing both biofilm and siderophores were able to sustain active growth, while the growth of the *epsA-O tasA* and *dhbA-F* mutants, unable to produce biofilm and siderophores, respectively, stopped. Based on these observations, we hypothesized that in the early stage, cell division is primarily supported by the consumption of cellular Fe stocks and that little or no Fe is taken up from the external medium. At mid stage, when Fe cellular stocks have been consumed, wild-type cells are able to support growth through the active acquisition of Fe from the medium, while mutants fail to do so and stop growing due to Fe limitation.

**FIG 3:**
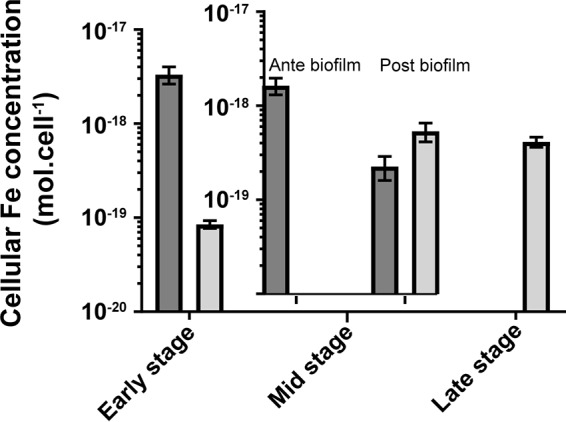
Acquisition of iron from the medium by B. subtilis during growth. Bars show intracellular concentrations of ^57^Fe (gray) and ^56^Fe (dark gray). Cells were grown at 30°C in MSgg medium supplemented with 10^−4^ M of ^57^Fe. Results are the means from the three biological replicates, and error bars represent standard deviations.

We tested this hypothesis by monitoring Fe acquisition before and after biofilm formation (mid stage) by using the pure stable ^57^Fe isotope, whose natural abundance is ∼2%. Cells preconditioned in the presence of naturally occurring Fe (∼92% ^56^Fe, ∼2% ^57^Fe) were inoculated in a growth medium containing pure ^57^Fe as a sole source of Fe (10^−4^ M ^57^Fe). This enabled us to track both the dilution of intracellular Fe, using the ^56^Fe signature of the preconditioned cells, and the acquisition of new Fe from the medium, using the ^57^Fe of the inoculation medium as a signature. As shown in Fig. 3, before biofilm formation (early stage and mid stage antebiofilm formation), both ^56^Fe and ^57^Fe intracellular concentrations decreased with growth. This confirms that in the early stage, there was no significant acquisition of Fe from the medium and that the intracellular Fe concentration primarily reflects the dilution of initial Fe stocks with cell division. After biofilm formation (mid-stage postbiofilm formation and late stage), the ^57^Fe concentration significantly increased, and ^57^Fe constituted the essential intracellular Fe at the end of growth. This result confirms that active acquisition of Fe from the medium happened after biofilm formation, and this acquisition was required to support the Fe needed for cell division. Overall, these observations confirm our hypothesis that the biofilm is required for active acquisition of Fe from the medium by B. subtilis in static MSgg cultures.

### Biofilm formation contributes to the siderophore-mediated uptake of Fe.

**FIG 4:**
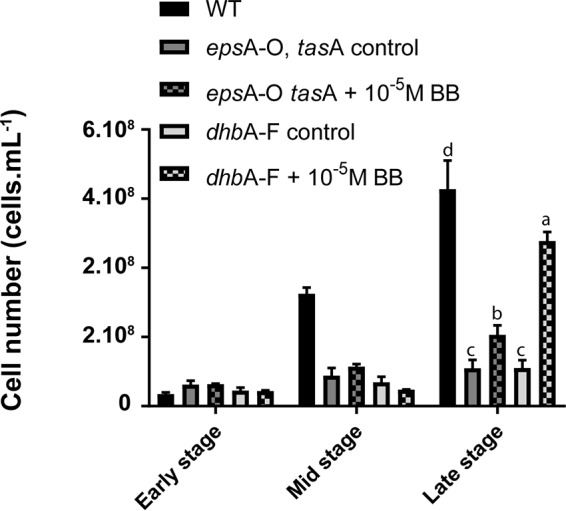
Effect of bacillibactin addition on the growth of B. subtilis WT and *dhbA-F* and *epsA-O tasA* mutants. Bars represent cell densities of *dhbA-F* and *epsA-O tasA* mutants in the presence (square patterns) and absence (solid patterns) of 10^−5^ M bacillibactin addition compared to that of the WT. Results are the means from three biological replicates, and error bars represent standard deviations. Strains were grown in at 30°C in MSgg medium supplemented with 10^−4^ M FeCl_3_. Lowercase letters indicate significant differences (*P* < 0.05).

While low Fe acquisition and impeded growth were predictable for the *dhbA-F* mutant, which lacks the essential siderophore for Fe uptake, the absence of significant uptake of Fe by the *epsA-O tasA* mutant was more surprising. Since bacillibactin production appears to be essential for sustained cell growth in MSgg with 10^−4^ M FeCl_3_, the growth impediment of the biofilm-deficient mutant (Fig. 2) might also have been caused by a lack of siderophore production. We observed that in the early stage of growth, the wild type and the *epsA-O tasA* mutant had the same bacillibactin production rates (∼10^−17 ^mol · cell^−1^, up to 13 h) (see Fig. S5). However, at mid stage (after 19 h), concomitantly with biofilm development in wild-type cells, bacillibactin production rates significantly decreased in the biofilm-deficient mutant while they remained roughly constant in wild-type cultures. This likely reflects the inability of Fe-starved B. subtilis *epsA-O tasA* cells to invest more resources in siderophore biosynthesis. To further investigate if the lower siderophore synthesis of *epsA-O tasA* at mid stage explained the Fe acquisition defect, we monitored the effect of bacillibactin addition on the growth of mutants deficient for biofilm formation (*epsA-O tasA* mutant) or siderophore production (*dhbA-F* mutant). Bacillibactin was added 13 h after inoculation at 10^−5^ M, corresponding to the concentration measured in wild-type cultures at the end of exponential growth (see Fig. S6). As expected, both biofilm formation and growth (Fig. 4; see also Fig. S7) of the *dhbA-F* mutant were restored roughly 9 h after the addition of bacillibactin. Growth rates after bacillibactin addition were comparable to the rates measured during exponential phase in the wild type (Fig. 4). However, growth of the *epsA-O tasA* mutant was only partially restored by the addition of bacillibactin (Fig. 4), confirming that the lack of siderophore production by this mutant is not the main reason for the growth arrest. Similarly, complementation of the *epsA-O tasA* mutant with an excess amount of bacillibactin-Fe complex restored growth to only approximately 30% (see Fig. S8). This result demonstrates that indeed, the growth defect of the *epsA-O tasA* mutant under our conditions was largely due to a defect in Fe acquisition and suggests the incapacity to efficiently recover Fe-bacillibactin. Taken together, these observations suggest that in liquid standing MSgg, the sole production of siderophores is insufficient to achieve the Fe acquisition required for normal growth (Fig. 2 and 4) and that biofilm formation significantly improves the retrieval of Fe-siderophore complexes from the medium. Since the *epsA-O tasA* mutant was only defective in extracellular matrix production and not in the genetic regulation leading to biofilm formation, it is very likely that these extracellular components play an important role in iron uptake.

### Complexation of Fe by catechol siderophores and Fe-siderophore complex acquisition efficiency.

**FIG 5:**
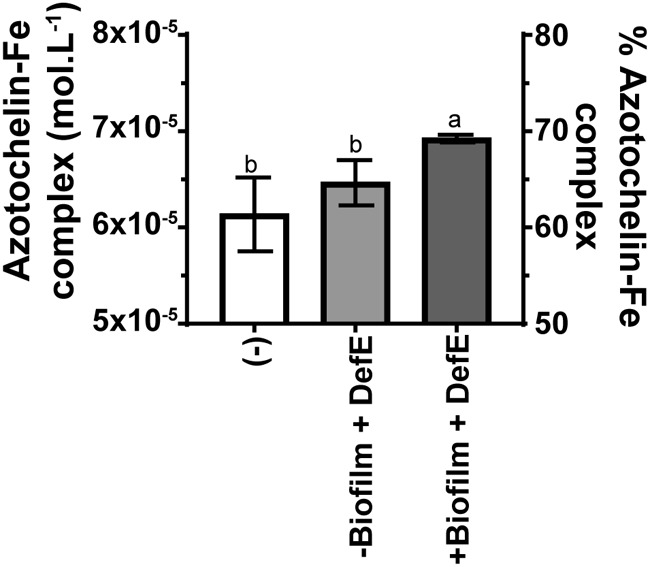
Complexation of Fe by the biscatechol siderophore azotochelin in the presence and absence of biofilm. Fe-azotochelin complex formation was monitored by UV-Vis spectrometry3 h after the addition of 10^−4^ M azotochelin. Complexation was assayed in MSgg medium (−), MSgg medium supplemented with 10^−5^ M DefE (−biofilm+DefE), and in the *dbhA-F* mutant cultured in MSgg medium supplemented with 10^−5^ M DefE (+biofilm+DefE).

It is known that kinetics are a major constraint to siderophore-mediated acquisition of Fe (10). We hypothesized that biofilm formation promotes Fe complexation kinetics, thus increasing Fe acquisition efficiency. To examine this, we measured Fe-catechol siderophore formation in the presence and absence of biofilm. Because of commercial constraints, we used the model siderophore azotochelin (a biscatechol produced by Azotobacter vinelandii), which possesses a structure analogous to that of bacillibactin. We also used a *dhbA-F* strain to which desferrioxamine E was added to induce the formation of a bacillibactin-free biofilm to ensure that bacillibactin would not interfere with Fe-azotochelin complex formation and measurement. As shown in Fig. 5, Fe complexation by bacillibactin was slightly improved in the presence of biofilm. While significant, this small difference (∼8% of the Fe-azotochelin complex formed after 3h; compare the plus biofilm column with the minus biofilm column in Fig. 5) is unlikely to solely explain the contrasting responses of the siderophore-deficient mutant and the biofilm-deficient mutant to bacillibactin addition.

## DISCUSSION

Our data show that in static culture, Fe homeostasis, i.e., a constant intracellular Fe concentration sustaining bacterial growth, is only reached in B. subtilis cells producing both siderophores and biofilms. Under our conditions, biofilm significantly improves the siderophore-mediated acquisition of Fe. This might be due to the presence of a densely packed community in the biofilm. Indeed, Völker and Wolf-Gladrow proposed that siderophore efficiency increases with cell density (24).

Because siderophores have a very high affinity for Fe, low concentrations of siderophores such as those found in soil (<10^−7^ M [25]) are, from a thermodynamic point of view, sufficient to promote Fe recruitment from insoluble oxides and other natural Fe complexes. Yet, slow Fe dissolution kinetics can constrain siderophore-mediated acquisition of Fe (10). We observed a slight increase in Fe complexation by catechol siderophores in the presence of a biofilm (Fig. 5), suggesting that it might provide a microenvironment promoting Fe complexation. Of note, exopolysaccharides have been shown to influence Fe speciation in biofilm compared to that in the supernatant (13), which might favor complexation by siderophores. In soils, where Fe is present in crystalized minerals (e.g., hematite and goethite) and in strong Fe-organic matter complexes, biofilm-promoted Fe complexation kinetics might play important roles in Fe acquisition and homeostasis. However, the gain of complexation efficiency was not solely responsible for the important increase in siderophore use efficiency we observed in the presence of biofilms.

It was recently demonstrated that biofilms of P. aeruginosa and Laminaria digitata can scavenge Fe, which can then be used by cells (13, 14). Trapping Fe hydroxides and oxides particles in the biofilm matrix likely prevents further condensation of oxides, which in return would facilitate Fe recruitment by siderophores. Also, many gradients have been reported in biofilms (e.g., pH, oxygen, metabolite concentration, and electric oscillation) (26, 27), which might promote the recruitment of Fe-siderophore complexes from the external medium. As presented in Fig. S6 in the supplemental material, bacillibactin concentrations were comparable in the medium and in the biofilm, but our analytical methods did not enable us to distinguish between apo-siderophores and metal-siderophore complexes. Thus, more research will be required to examine how biofilm properties (physical structure and chemical composition) might promote Fe-siderophore acquisition. Nonetheless, our data support the theory that the biofilm provides microenvironmental conditions stimulating an efficient recruitment of Fe from hydroxides, oxides, and other natural Fe complexes by siderophores.

Finally, our study highlighted the contrasting efficiencies in siderophore-mediated Fe acquisition between shaken and static cultures. In the presence of similar Fe concentrations, the bacterial growth rates of wild-type and *epsA-O tasA* mutant cells were similar in shaken cultures (Fig. S4), while in static cultures, the absence of biofilm resulted in severely impeded growth by the *epsA-O tasA* mutant, even in the presence of siderophore addition (Fig. 2 and 4). Static cultures face challenges, such as the distance between cells and Fe sources, the diffusion of siderophores and Fe-siderophore complexes in the medium, that shaken cultures do not encounter. Many studies on Fe homeostasis and siderophore-mediated acquisition of Fe by soil bacteria have been performed in shaken cultures (28, 29). Thus, the importance of the interplay between siderophore production and extracellular matrix production to support efficient Fe acquisition and Fe homeostasis might have been overlooked. Considering that static growth in a biofilm is a highly relevant growing state for many soil bacteria, more research on metal acquisition and homeostasis by model soil bacteria in static cultures is required to draw a more comprehensive picture of the role of biofilm production in siderophore use efficiency and metal homeostasis.

## MATERIALS AND METHODS

### Strains and mutant constructions.

The Bacillus subtilis strains used in this study are listed in Table S1 in the supplemental material. B. subtilis strain NCIB3610 was used as the wild type due to its capacity to form a robust biofilm, and the mutants were constructed in this genetic background. The *dhbA-F*::*erm* deletion mutant was constructed using the long flanking homology PCR technique. The primers used were as follows (5′→3′): P155, ATG CCA ACA GCC CAA TCG AA; P156, GAG GGT TGC CAG AGT TAA AGG ATC CTT GGG CAG CCC CTG TTA TAA A; P157, CGA TTA TGT CTT TTG CGC AGT CGG CTG GCT CAA ATC GGC AAG GTT; and P158, CGT ATT ACA GGT GCG CCA TCA A. PCR products for gene deletions were introduced in B. subtilis strain 168 by natural competence (30). The genetic deletion was then transferred to the strain NCIB3610 or other appropriate mutant strains by SPP1-mediated generalized transduction (31).

### Preculture and biofilm formation.

To prepare the inoculum for biofilm formation, B. subtilis cells were precultured from glycerol stocks for 18 h on LB (Lennox broth) agar plates at 37°C. Colonies were harvested from the LB plates and suspended in 50 ml of MSgg medium without iron to generate an inoculum at an optical density at 595 nm (OD_595_) of 1 ± 0.05. Of note, these precultures achieved cellular Fe concentrations significantly higher than metabolic minimum needs. For all experiments, the inoculum was prepared in LB, allowing the cells to reach 4.1 × 10^−18^ mol Fe · cell^−1^. The biofilm medium used throughout this study was MSgg without iron (5 mM potassium phosphate buffer [pH 7], 0.1 M MOPS [morpholinepropanesulfonic acid] [pH 7], 2 mM MgCl_2_, 0.05 mM MnCl_2_, 0.001 mM ZnCl_2_, 0.002 mM thiamine, 0.5% glycerol, 0.5% glutamate, 0.7 mM CaCl_2_) (32). Milli-Q deionized water was used to prepare the medium. Prior to the medium preparation, the glassware was washed for 24 h with a 10% solution of HCl (trace metals reagent) and rinsed three times with Milli-Q water to prevent iron contamination of the MSgg. When indicated, iron was added in the medium from a sterile solution of FeCl_3_, and the final FeCl_3_ concentration is specified in the legend of each figure. To induce biofilm formation, the wells of sterile 6-well plates were filled with 4.8 ml of MSgg medium with the selected amount of FeCl_3_ (10^−4^ M) and inoculated with 150 μl of the B. subtilis inoculum suspension (time zero). Incubations were performed at 30°C (30 h, static).

### Cells isolation and analysis.

The methodology and controls for cell isolation and analysis, including the measurement of growth rates, are detailed in the methods in the supplemental material.

### P*_tapA_-yfp* reporter and flow cytometry analysis.

The analysis of biofilm formation was performed using flow cytometry and an *amyE*::P*_tapA_-yfp* reporter, which consists of a fusion between the promoter of the biofilm-induced *tapA* matrix gene and the YFP (yellow fluorescent protein)-encoding gene. When biofilm formation is triggered, a subpopulation of cells will produce the biofilm matrix and thus express the YFP, which can be detected by flow cytometry (33, 34). The increase in the percentage of YFP-positive cells correlates with the increase in the biofilm (pellicle) weight (see Fig. S9). For flow cytometry analysis, cells in GTE (described in the methods section of the supplemental material) were sonicated with a Q125 sonicator (power 20%, 10 pulses of 1 s with 1-s pauses) and diluted in phosphate-buffered saline (PBS) to obtain an event rate of 300 to 800 events per second. Data collection was performed with a BD FACSJazz using a 488-nm laser. Data analysis was performed using BD software; the gates were created such that the first one encompasses only the fluorescent population while the second encompasses the population of all bacteria, which was set to 20,000 events.

### Elemental analysis.

Cells were digested on an SCP science Digiprep Jr with 1 ml of nitric acid (trace metals grade; Fisher Chemical) at 65°C for 45 min. After digestion, each tube was filled to 10 ml with Milli-Q water. Samples were analyzed for phosphorus and metal contents on an inductively coupled plasma mass spectrometer (ICP-MS XSeries 2; Thermo Scientific) as previously described (35).

### Siderophore extraction and quantification.

Bacillibactin was quantified as described in reference 36. Briefly, bacillibactin was extracted, purified by solid-phase extraction (HLB cartridges; Waters), and quantified using ultra-high-pressure chromatography coupled to a triple-Quad mass spectrometry (Acquity UPLC-TQ-MS, XEVO; Waters Corporation, Milford, MA). More details on bacillibactin extraction, purification, and analysis are presented in Tables S2 to S4 and the methods in the supplemental material.

### Fe complexation by the catechol siderophores.

Complexation of freshly precipitated Fe (10^−5^ M FeCl_3_) by bacillibactin (10^−5^ M) in water was monitored over time by UV-visible (UV-Vis) spectrometry (Genesys 10S; Thermo Scientific). Experiments on Fe complexation by a catechol siderophore in the presence of biofilm were performed at a high concentration of ligand (10^−4^ M) to overcome the spectral interferences of the biofilm matrix. Due to the limited commercial availability of bacillibactin, we used azotochelin, a biscatechol from Azotobacter vinelandii, as a model catechol siderophore. Azotochelin was synthetized as described previously (37, 38). The B. subtilis *dhbA-F* mutant (deficient in siderophore production) was grown in the presence of 10^−4^ M FeCl_3_ and 10^−5^ M desferrioxamine E (DefE; Cedarlane, Burlington, Ontario, Canada) to obtain a catechol siderophore-free biofilm. After biofilm establishment (22 h after inoculation), 10^−4^ M azotochelin was added to the medium, and the formation of Fe-azotochelin complex was monitored by UV-Vis spectrometry (Genesys10s; Thermo Scientific).

### Statistical analysis.

Statistical analyses were performed using GraphPad Prism version 7. The comparison shown in Fig. 5 was performed using a one-way analysis of variance (ANOVA; *P* < 0.05).

Comparisons shown in Fig. 2 and 4 were determined in at least triplicate using Student’s *t* tests (*P* < 0.05).

## Supplementary Material

Supplemental file 1
