# Supplementary material for: Iron Homeostasis in Bacillus subtilis Requires Siderophore Production and Biofilm Formation
Source: Appl Environ Microbiol. 2019 Jan 23;85(3):e02439-18. doi: 10.1128/AEM.02439-18 (PMC6344612; doi:10.1128/AEM.02439-18)
Supplement: Supplemental file 1 [file 14a4ada801d815d59be942967e3e5675_AEM.02439-18-s0001.pdf]

Biofilm formation responds to iron limitation and promotes the siderophore-based iron acquisition in *Bacillus subtilis*

Supplementary Material

Adrien Rizzi[1,2], Sébastien Roy[2], Jean-Philippe Bellenger[1§], Pascale B. Beauregard[2§]

[1]Département de chimie, Faculté des Sciences, Université de Sherbrooke, Sherbrooke, Canada

[2]Centre SÈVE, Département de biologie, Faculté des Sciences, Université de Sherbrooke, Sherbrooke, Canada

[§]Correspondence:

P. B. Beauregard, Département de biologie, Université de Sherbrooke, 2500 boulevard de l'Université, J1K 2R1, Sherbrooke (Québec), Canada. email : pascale.b.beauregard@usherbrooke.ca

J. P. Bellenger, Département de chimie, Université de Sherbrooke, 2500 boulevard de l'Université, J1K 2R1, Sherbrooke (Québec), Canada. email : j-p.bellenger@usherbrooke.ca

SUPPLEMENTARY METHODS

Cells isolation and preparation for flow cytometry and elemental analysis

The cell isolation and preparation protocol was elaborated to fit several criteria:

1. Cells need to be fixed, in order to keep the YFP fluorescence from the biofilm reporter intact.
2. Cells need to be isolated from the biofilm matrix, since exopolysaccharides from the matrix are likely metal chelators that would impact the evaluation of metal content.
3. Cells need to be submitted to repeated oxalate-EDTA washes in order to get rid of Fe precipitated on the cell surface(1).

Consequently, the following protocol was established:

At regular time intervals during growth in multiwall plates, cells were harvested in 15mL vials and pelleted by centrifugation (Beckman Coulter$_{TM}$ Adventi centrifuge J-25I with JLA 16,250 rotor, 6500 xg, 20°C, 7 min). The cell-containing pellets are then immediately suspended in 1mL of a 4% paraformaldehyde (PF) solution (4% paraformaldehyde in PBS) and incubated 7 minutes at room temperature. This step allows fixation of the cells, and conservation of the fluorescent proteins integrity. The fixed cells were then pelleted by centrifugation (6500 xg, 20°C, 7 min). The pellet was re-suspended in 1mL oxalate/EDTA solution (0.1 M/ 0.05 M) and incubated at room temperature for 7 minutes, both to remove external metals that could be trapped in the biofilm matrix and to wash the leftover paraformaldehyde. Cells were again pelleted by centrifugation (6500 xg, 20°C, 7 min), and then re-suspended in 5 mL of NaCl 0.5 M. The NaCl suspension of cells was separated in two, in order to do parallel analysis on the same sample: flow cytometry (A) and elemental analysis (B). In the case where only the elemental analysis is performed, the whole 5mL of cells in NaCl is treated according to (B).

(A) 1 mL of cells of the cells in NaCl suspension was centrifuged on a Fisher Scientific AccuSpin Micro17 (13,300 xg, 2 min). The pellet was the suspended in 1 mL of GTE solution (50 mM Glucose, 10 mM EDTA, 20 mM Tris pH8) and stored at 4°C for a maximum of 3 days before flow-analysis.

(B) 4 mL of cells in NaCl was sonicated on a Q125-Sonicator (power 20%, 10 pulses of 1 sec with 1 sec pause) to detach the clumped cells. Then, NaOH was added to a final 0.1M concentration to help solubilize the biofilm matrix at RT for 5min (adapted from(2). Cells were then separated from the biofilm matrix, now soluble, by centrifugation (6500 xg,

20°C, 7 min). Pelleted cells were then washed once with 1mL oxalate/EDTA (0.1 M/ 0.05 M), pelleted (6500 xg, 20°C, 7 min), and washed again with 1mL of a diluted oxalate/EDTA solution (0.025M/ 0.0125M) solution. Cells were finally pelleted by centrifugation (6500 xg, RT, 7 min), and the pellets were then store at 4°C until elemental analysis.

Controls performed for method validation: cell lysis

Controls were performed to validate that the various steps during cells isolation and preparation did not affect the cellular metal content. First, we evaluated if sonication and/or NaOH treatment caused cell lysis and leaking of metals. These controls were performed with the biofilm mutant *epsA-O tasA* to avoid a possible metal-chelating effect of the biofilm matrix, which would bias the results. Briefly, 9 replicates were treated as followed

(A) 3 replicates were fixed with PF, and washed twice with oxalate-EDTA.
(B) 3 replicates were fixed with PF, washed once with oxalate-EDTA, resuspended in NaCl and sonicated as describe above, and washed again with oxalate-EDTA.
(C) 3 replicates were fixed with PF, and washed once with oxalate-EDTA, resuspended in NaCl, treated with NaOH, and washed again with oxalate-EDTA

All 9 replicates were then digested on a SCP Science Digiprep Jr with 1mL of nitric acid at 65°C for 45min. After digestion, each tube was filled at 10mL with Milli-Q water, and samples were analyzed for phosphorus, manganese, magnesium and iron content on an inductively-coupled-plasma mass spectrometer (ICP-MS; Thermo Scientific XSerie2). All samples showed similar amount of metals (Fig S10). Since there was no difference between the fixed (A), fixed and sonicated (B), fixed, sonicated and NaOH treated cells (C), we conclude that the treatments do not cause cell lysis.

Controls performed for method validation: matrix solubilization

Controls were performed to examine if our treatments efficiently get rid of the biofilm matrix and of a putative biofilm matrix-chelator effect. 9 replicates of WT cells were grown in MSgg during 25h to obtain a mature biofilm, and collected by centrifugation. At this step, 3 supernatants were kept as is (M1). Cells were then fixed with PF and pelleted; after this 3 supernatants (S1) and 3 pellets were kept (T1). Remaining 6 cell pellets were resuspended in NaCl and sonicated as described, after which 3 replicates were kept (T2). 2 mL NaCl and 500uL NaOH 1M were added to the 3 last replicates, and cells were collected by centrifugation. After this centrifugation step, supernatants (S3) and cell pellets (T3) were kept. Each sample was observed by microscopy for cell-clumping (indicative of leftover biofilm) and tested for chelating properties by CAS and

Arnow assay. The table below indicates that after the whole treatment, the cell pellet (T3) show no sign of biofilm matrix left, since cells are not clumped and there is no metal-chelation activity.

Samples	CAS	Arnow	Cell clumping
M1	+++	+++	NA
S1	++	++	NA
T1	+	+	++
T2	+	-	+
S3	-	-	NA
T3	-	-	-

Cell quantification

Cells were grown in MSgg at 30°C during 28 hours as previously described. Every 3 hours during growth, triplicates cell samples were collected by centrifugation on a Beckman Coulter$_{TM}$ centrifuge J-25I with JLA 16,250 rotor (6500g, 20°C, 7min). The cell-containing pellets were treated as describe previously for elemental analysis, and the final resuspension was performed in 5 mL NaCl. 1 mL of this cell suspension was used to determine cell with a Petroff-Hausser device on an inverse fluorescence microscope Zeiss Z1-Observer using DIC illumination (40x); each replicate was measured 3 time. The leftover cell suspension (4 mL per replicate) was digested on a SCP science Digiprep Jr with 1mL of nitric acid (trace metal grade, Fisher Chemical) at 65°C for 45 min. After digestion, each tube was filled at 10 mL with Milli-Q water, and analyzed for phosphorus on an inductively-coupled-plasma mass spectrometer (ICP-MS; Thermo Scientific, XSeries2) as previously described (3)**.** Cell number was plotted in function of intracellular phosphorus (Figure S1), and shows a strong linear correlation.

Bacillibactin and DHBA extraction and quantification

Bacillibactin and DHBA extraction from supernatant. BB and DHBA in the supernatant were analyzed after a sample purification. Briefly, 1 mL of the supernatant was passed thought an Oasis HLB solid phase extraction cartridge and then reconstituted in 1 mL of methanol. The biomass (cellule and matrix) was withdrawn and 30 mg of NaCl and 1 mL of a mixture of methanol and acetonitrile (1:1 v/v) with 0.20% of formic acid was added to the sample. After 3 times 5 min of ultrasonic and vortex agitation, the supernatant was then centrifuged at 4600 rpm for 15 min at 4 °C and the supernatant was filtered through a 0.22 µm PTFE filter before analysis

BB et DHBA quantification: The analyses of BB and DHBA were performed using a positive electrospray ionization (ESI-) source in Multi-Reaction-Monitoring mode on an Acquity UPLC XEVO TQ mass spectrometer (Waters Corporation, Milford, MA) equipped with an Acquity UPLC HSS-T3 column (100 mm × 2.1 mm, 1.8 µm) and a fritted 0.2 µm prefilter. The solvent flow rate was set to 0.40 mL min^{-1} and the column temperature was kept at 40 º C. The sample volume injected was 5 µL. The mobile phase was 0.20% formic acid/water (A) and 0.20% formic acid/methanol-acetonitrile (80-20 v+v) (B). The elution gradient started with 5% of eluent B, increasing to 100% in 4 min and then back to initial conditions in 2 min for a total run time of 8 min. The optimized parameters were obtained by direct infusion of BB or DHBA analytical standard solutions at 10 µg mL^{-1} as follows: desolvation gas (nitrogen) at 800 L h^{-1}; cone gas (nitrogen) at 50 L h^{-1}; collision gas (nitrogen) at 0.22 mL min^{-1}; capillary voltage 2.5 kV; source temperature, 150 º C and desolvation temperature 550 º C. Two daughter traces (transitions) were used. The most abundant transition was used for quantification, whereas the second most abundant was used for confirmation. MS parameters are available in supporting information Table S2, S3, and S4.

SUPPLEMENTARY TABLES

Table S1 Strains used in this study

Strain	Genotype	Sources/Reference
NCIB3610	WT	Lab Stock
P_{tapA}-*yfp*	*amyE::*P_{tapA}-*yfp (spec)*	(4)
epsA-O tasA	*epsA-O*::*tet* ,*tas*A::*erm*	Lab Stock
dhbA-F	*dhbA-F::erm*	This study

Strains are all derivatives of *B. subtilis* 3610. Antibiotics: spectinomycin (spec), tetracycline (tet), erythromycin / lincomycin (erm)

Table S2: Chromatographic elution gradient used for BB and DHBA separation and identification

Time (min)	Flow (mL.min^{-1})	A%	B%
Initial	0.40	95	5
1	0.40	95	5
4.00	0.40	0	100
6.00	0.40	0	100
7.00	0.40	95	5
9.00	0.40	95	5

Table S3: Source parameters used for the determination of BB

**Capillary**	2.5 Kv	**LM 1 resolution**	2.8
**Extractor**	3 V	**HM 1 resolution**	14.7
**Source Temp**	150 ºC	**Ion energy 1**	0.5
**desolvatation Temp**	550 ºC	**LM 2 resolution**	3
**Cone gas flow**	50 L/h	**HM 2 resolution**	15
**Desolvation gas flow**	800 L/h	**Ion energy 2**	0.5
**Collision gas flow**	0.22 mL/min	**Multiplier**	504

<u>Table S4:</u> Mass spectrometry parameters used for the quantification of BB and DHBA.

**Active Substance**	**Transition 1 : Quantification**		
	**Parent > T1 (m/z)**	**Cone (V)**	**Coll (V)**
**BB**	881 > 249	48	44
**DHBA**	153 > 109	25	21

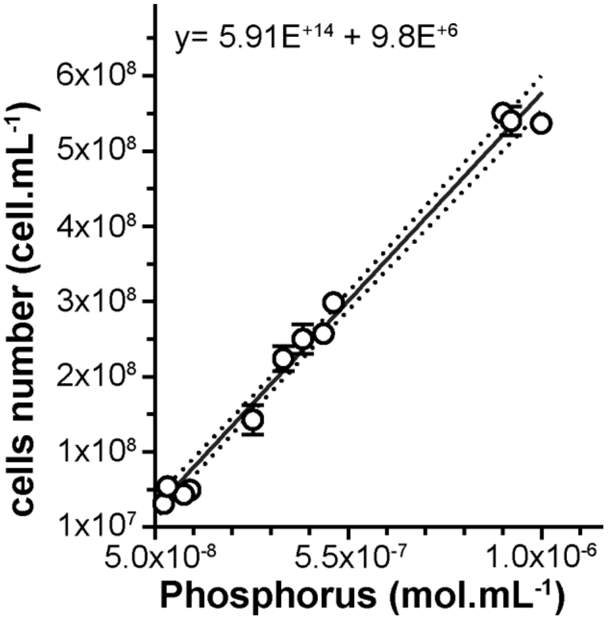

Figure S1 Linear regression between intracellular phosphorus ($mol.mL^{-1}$) measured by ICP-MS and *B. subtilis* cell density measured by microscopy on Z1-Observer with a Petroff Hausser chamber ($cell.mL^{-1}$). The black line represents linear regression with a $r^2 = 0.98$. The dashed lines represent 95% CI.

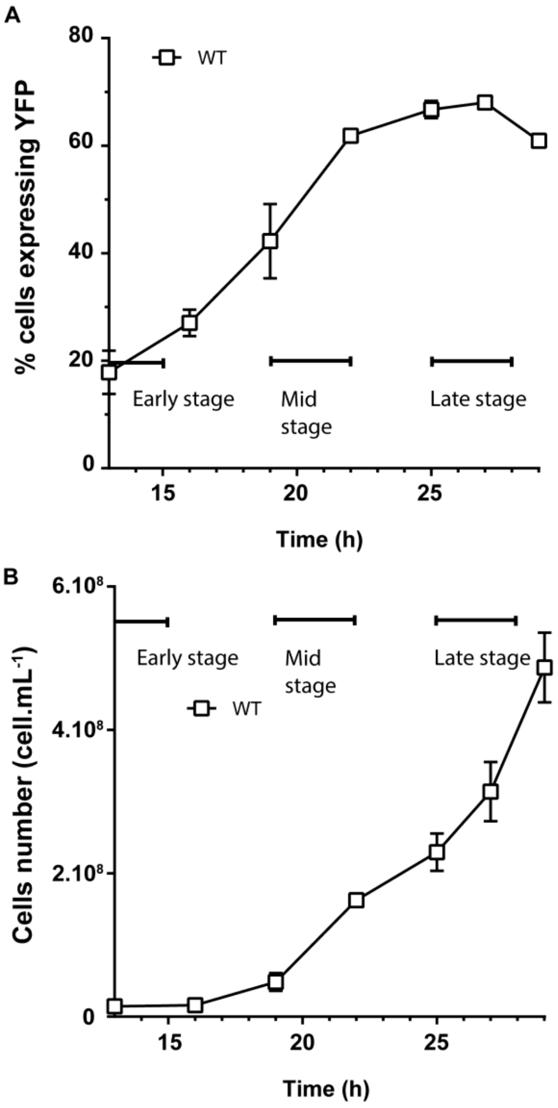

Figure S2 Biofilm induction (A) and growth (B) of *B. subtilis* 3610 P_{tapA}-*yfp* grown at 30°C in a MSgg medium supplemented with 10^{-4} M $FeCl_3$ (n = 3). Early stage is defined before Biofilm apparition, and late stage after biofilm is well established.

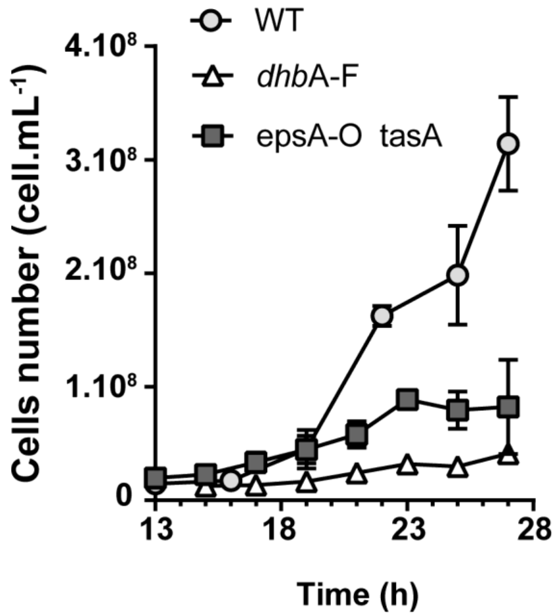

Figure S3 Growth of *B. subtilis* WT (grey circle), mutant *epsA-O tasA* (Dark-grey squarel) and mutant *dhbA-F* (white triangle) in a MSgg medium supplemented with 10^{-4} M $FeCl_3$ at 30°C. Phosphorus quantification was used as a proxy for cell quantification (Fig S1). Results are the mean of the three biological replicates and error bars represent standard deviation.

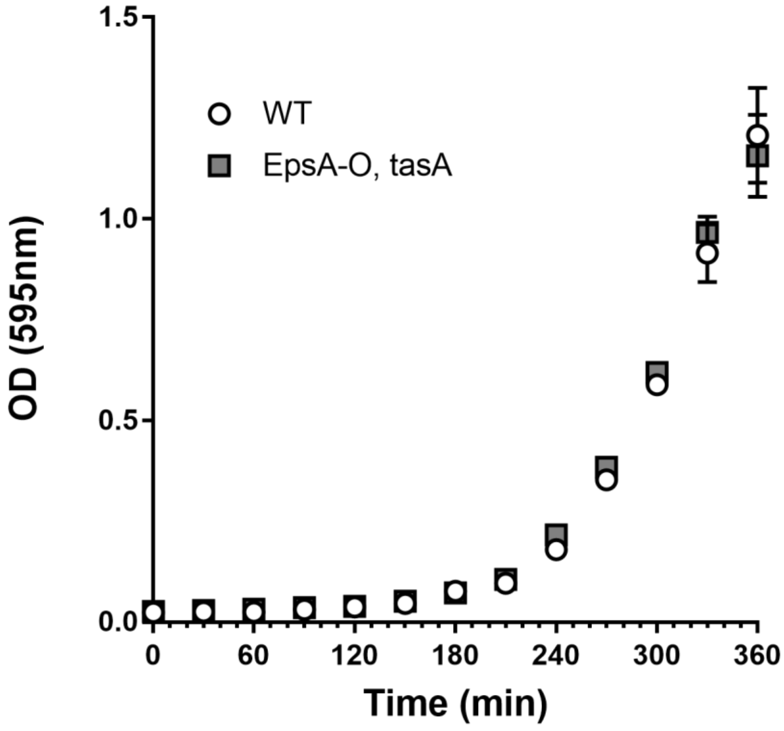

Figure S4. Growth of *Bacillus subtilis* WT and *eps*A-O *tas*A mutant in shacked (150 rpm) liquid MSgg medium (10^{-4}M $FeCl_3$ and at 30°C).

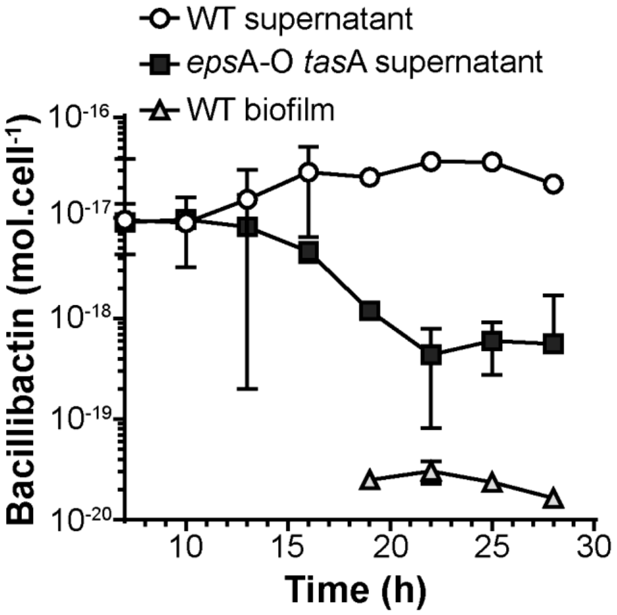

Figure S5. Production of bacillibactin (mol.cell^{-1}) by *B. subtilis* WT and *epsA-O tasA* mutant during growth in MSgg supplemented with 10^{-4} M $FeCl_3$ at 30°C.

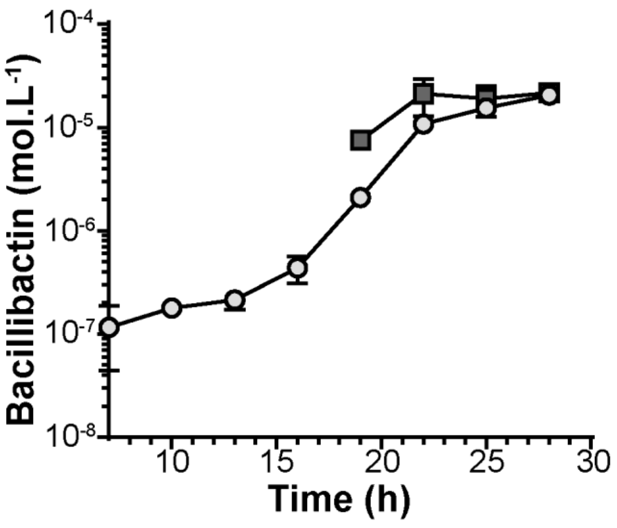

Figure S6. Concentration of bacillibactin (mol.L^{-1}) in supernatant (circles) and biofilm (squares) of *B. subtilis* wildtype during growth in a MSgg medium supplemented with 10^{-4} M $FeCl_3$ at 30°C.

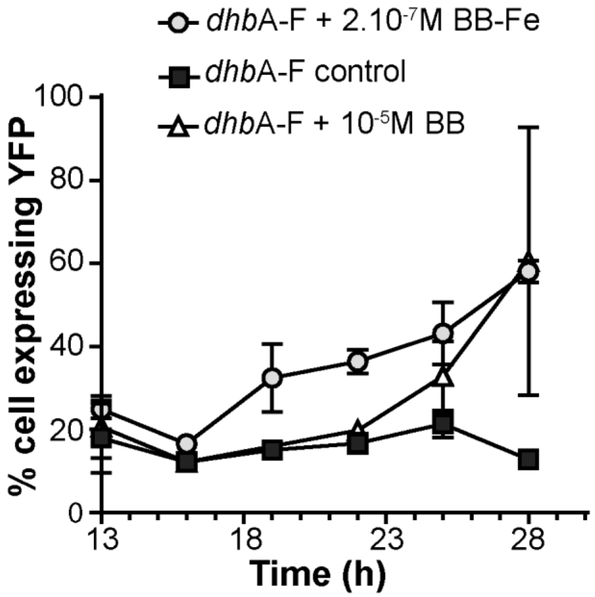

Figure S7 Induction of biofilm by the *dhba-F* mutant in presence of 10^{-5} M bacillibactin (white triangles), of 2 x 10^{-7} M bacillibactin-Fe (gray circles) or in absence (black squares) of bacillibactin. The strain was grown in MSgg supplemented with 10^{-4} M $FeCl_3$ at 30°C.

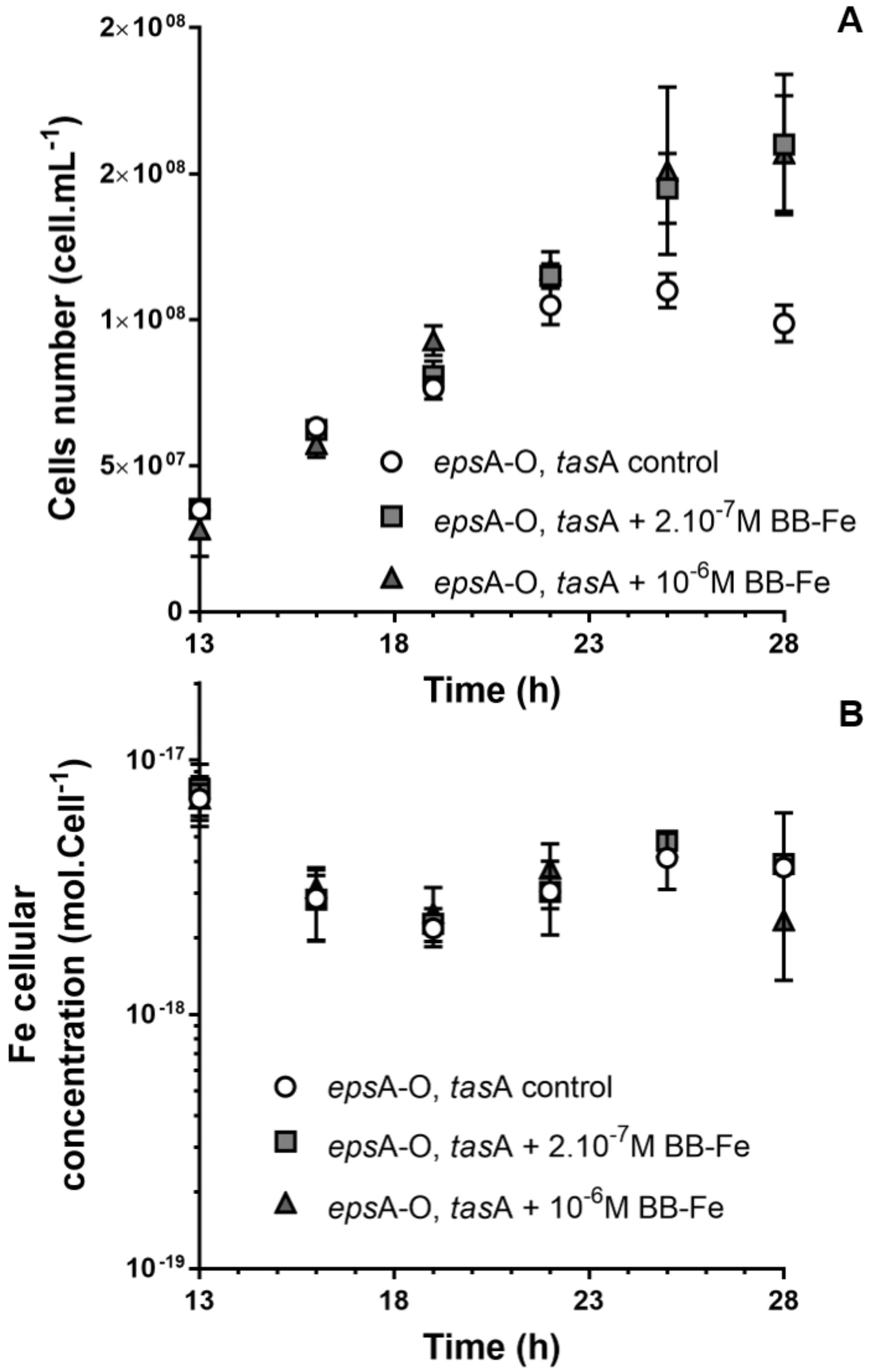

Figure S8. Growth (A) and intracellular iron concentrations (B) of *eps*A-O *tas*A mutant in presence (squares and triangles) and absence (circles) of Fe-bacillibactin complex

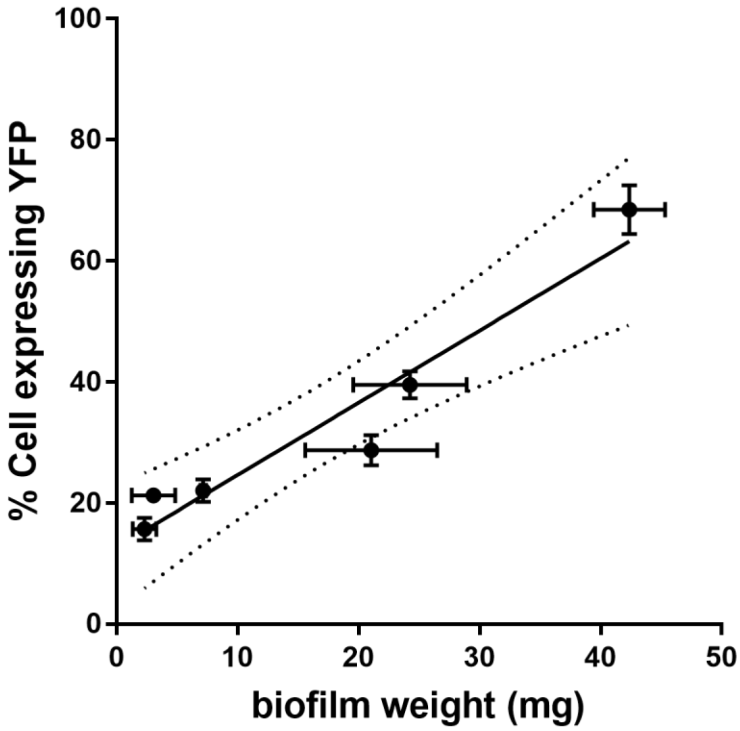

Figure S9 Linear correlation between biofilm dry weight and expression of the P_{tapA}-*yfp* reporter in MSgg supplemented with 10 $^{-4}$M $FeCl_3$ at 30°C. The black line represents linear regression with a r^2 = 0.93.The dashed lines represent 95% CI. Samples were collected at regular time interval during biofilm development (from 13 h to 28 hours).

References

1. Tang D, Morel F, M. M. 2006. Distinguishing between cellular and Fe-oxide-associated trace elements in phytoplankton 98:18–30.
2. Jachlewski S, Jachlewski W., Linne U, Bräsen C, Wingender J, Siebers B. 2015. Isolation of extracellular polymeric substances from biofilms of the thermoacidophilic archaeon Sulfolobus acidocaldarius 3:1–11.
3. Darnajoux R, Constantin J, Miadlikowska J, Lutzoni F, Bellenger JP. 2014. Is vanadium a biometal for boreal cyanolichens ? New Phytol 765–771.
4. Vlamakis H, Aguilar C, Losick R, Kolter R. 2008. Control of cell fate by the formation of an architecturally complex bacterial community 945–953.
